# Microbiome diversity of cotton aphids (*Aphis gossypii*) is associated with host alternation

**DOI:** 10.1038/s41598-021-83675-2

**Published:** 2021-03-04

**Authors:** Yan-jie Ma, Hao-peng He, Hai-meng Zhao, Yi-dan Xian, Hui Guo, Biao Liu, Kun Xue

**Affiliations:** 1 College of Life and Environmental Sciences, Minzu University of China, Beijing, 100081 China; 2 Nanjing Institute of Environmental Sciences, MEP, Nanjing, 210042 China

**Keywords:** Symbiosis, Entomology

## Abstract

Aphids are infected by a series of bacteria that can help them survive on specific host plants. However, the associations between aphids and these bacteria are not clear, and the bacterial communities in many aphid species are poorly characterized. Here, we investigated the bacterial communities of cotton aphids (*Aphis gossypii*) on 2 representative winter host plants and transferred to 3 summer host plants by 16S rDNA sequencing using the Illumina MiSeq platform. Our results revealed that the bacterial communities varied among cotton aphids on hibiscus, cotton aphids on pomegranate, cotton aphids on cotton transferred from hibiscus, cotton aphids on muskmelon transferred from hibiscus, cotton aphids on cucumber transferred from hibiscus,. The diversity and richness of the bacterial communities were significantly higher in aphids on muskmelon and aphids on cucumber than in the other treatments. There were two main factors influencing the distribution of internal bacterial OTUs revealed by principal component analysis, including the differences among Punicaceae, Malvaceae and Cucurbitaceae. There were 28 bacterial communities with significant differences between two arbitrary treatments, which could be grouped into 6 main clusters depending on relative abundance. Moreover, our results indicated that in addition to the obligate endosymbiont *Buchnera*, with a dominant position (> 52%), *A. gossypii* also harbored 3 facultative endosymbiotic bacteria (*Serratia*, *Arsenophonus*, and *Wolbachia*) and 3 possibly symbiotic bacteria (*Acinetobacter*, *Pantoea*, and *Flavobacterium*). There were several correspondences between the symbiotic bacteria in cotton aphids and the specific host plants of the aphids. This study provides a better understanding of the interactions among symbiotic bacteria, aphids and host plants, suggesting that the selection pressure on aphid bacterial communities is likely to be exerted by the species of host plants.

## Introduction

Interactions between polyphagous herbivorous insects and microbes are ubiquitous in nature. Microbial symbionts of insect hosts have emerged as key contributors to many biological processes and evolutionary adaptation^1,2^. For instance, these symbionts can regulate the nutrient metabolism^3,4^ and reproductive metabolism of host insects^5^, assist insects in resisting biological and abiotic stresses^6,7^, and improve insects’ resistance to chemical pesticides and adaptability to host plants^8^. These microorganisms and their host insects have established a close mutualistic relationship over the course of long-term coevolution^9^.

The cotton aphid (*Aphis gossypii* Glover) is a global agricultural pest that is capable of breeding on over 600 species of plants in tropical, subtropical and temperate regions^10^. The hosts of cotton aphids can be divided into winter hosts and summer hosts. Winter hosts mainly include pepper, buckthorn, pomegranate, hibiscus, selfheal, plantain and others. Summer hosts include plants from Malvaceae, Cucurbitaceae, Leguminosae and Compositae, among which cotton and melon are the most important hosts^11^. In general, there are 2 crucial migratory flights that allow cotton aphids to change host plants in order to adapt to seasonal changes. One migratory flight involves the transfer from winter hosts to summer hosts in the early spring in a parthenogenetic form. The other involves the transfer from summer hosts to winter hosts in late autumn in a sexually reproductive form. The species overwinters in the form of eggs^12^. In addition, cotton aphids may change their host to seek better living conditions in adverse environments^13^.

Cotton aphids have an obvious preference for host plants^14^, which makes it difficult and time-consuming for aphids with a specific preference to transfer to another host plant for development and reproduction under natural conditions. This phenomenon is called host specialization^15^. Nevertheless, host alternation (cotton aphids transferred from the original host to another non-original host and establish a stable population) can be accelerated under laboratory conditions. *Nilaparvata lugens* can complete this transfer within just 10 generations of laboratory rearing^16^. Previous studies have shown that the formation of aphid host specialization is associated with differences in host plants and aphids. On the one hand, aphids can identify their hosts by plant pheromones or stylet penetration behaviors^17,18^. On the other hand, plants such as *Cucumis* contain pyrazoles that are toxic to pea aphids but do not affect cotton aphids^19^. Therefore, only cotton aphids can specialize on such species.

Recent studies have shown that host specialization is also associated with facultative endosymbionts. Ferrari et al. found that six facultative endosymbionts were nonrandomly distributed and that geography contributed little to the evolution of specialization and ecological speciation of *Acyrthosiphon pisum* on different hosts, which is collected from eight legume genera in England and Germany^20^. Based on a phylogenetic and ecological distribution study of 1104 pea aphids, Henry et al. concluded that facultative endosymbionts formed a horizontal gene pool, which influenced the adaptation and distribution of their insect hosts^21^. Vorburger et al. found that aphids from summer hosts of *Cirsium* and *Chenopodium* exhibited differences in their frequencies of infection by particular endosymbionts^22^. Similar phenomena were found in the facultative endosymbionts of *Aphis craccivora*^23^.

To date, although some studies have been conducted on the relationships between endosymbionts and aphids, there are few studies on the alteration of endosymbionts of cotton aphids during host transformation. Therefore, our objectives were to (1) characterize and compare the internal bacterial communities of cotton aphids from 2 winter host plants before and after transferring them to 3 summer host plants and (2) determine the distribution of several major endosymbiotic bacteria on these 5 host plants.

## Results

### OTUs in aphids on different host plants

**Figure 1:**
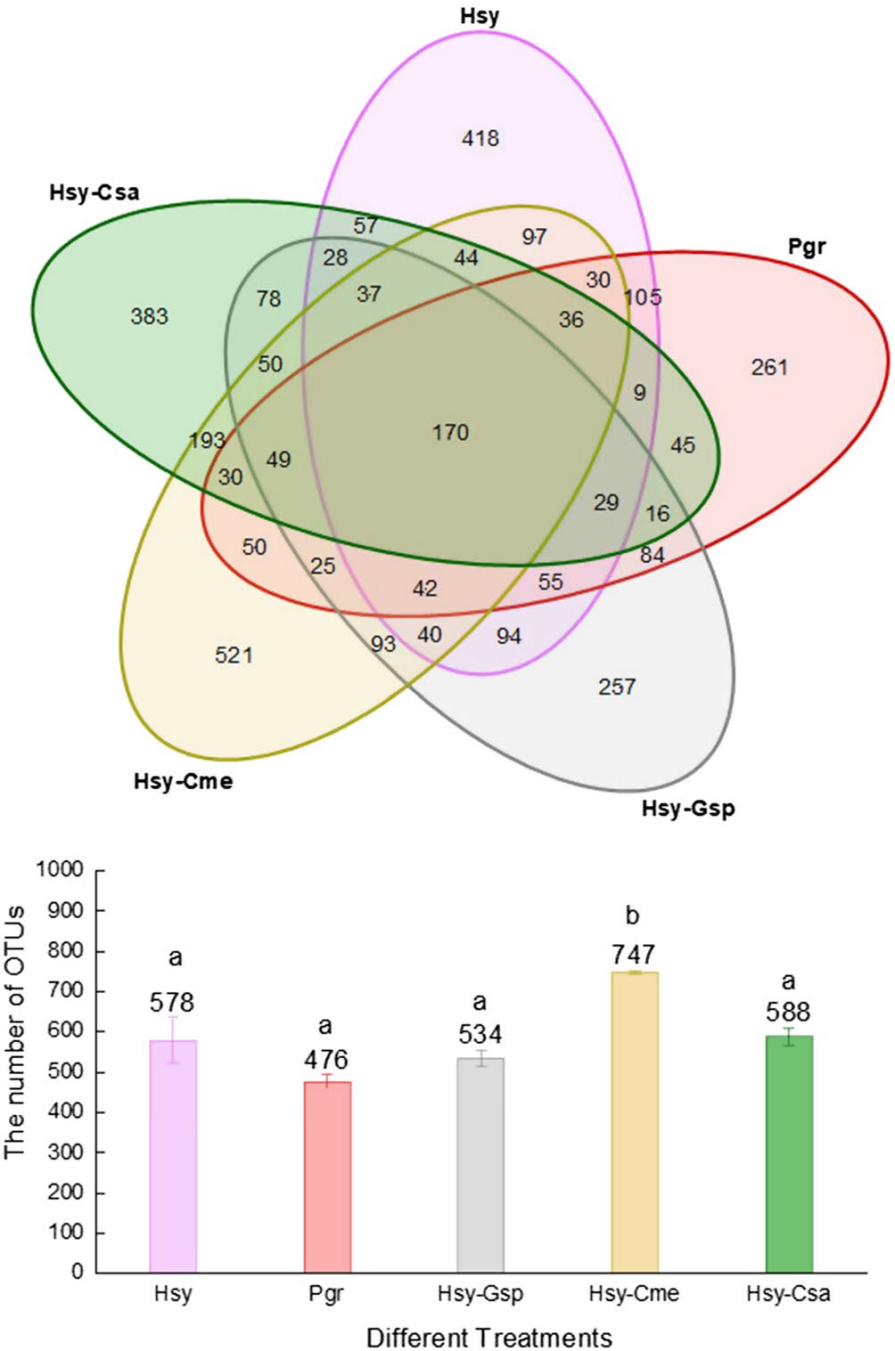
Venn diagram and bar chart of OTUs in Hsy, Pgr, Hsy-Gsp, Hsy-Cme and Hsy-Csa. The numbers in overlapping regions represent the number of OTUs shared by treatments. The numbers in nonoverlapping regions represent the number of unique OTUs in each treatment. The numbers above the bars represent the average number of OTUs in different treatments. Different lowercase letters above the bars indicate significant differences in the number of OTUs among treatments (P < 0.05).

The number of OTUs corresponding to each group and a Venn diagram of the five groups are shown in Fig. 1. The numbers of internal bacterial OTUs (IBOs) in Hsy (cotton aphids on hibiscus), Pgr (cotton aphids on pomegranate), Hsy-Gsp (cotton aphids on cotton transferred from hibiscus), Hsy-Cme (cotton aphids on muskmelon transferred from hibiscus) and Hsy-Csa (cotton aphids on cucumber transferred from hibiscus) were 577.67 ± 56.84, 475.67 ± 16.19, 533.67 ± 20.58, 747.00 ± 3.51, and 588.00 ± 21.17, respectively. The number of IBOs in Hsy-Cme was significantly larger than that in the other treatments. The smallest number of IBOs was observed in Pgr. A total of 170 IBOs appeared in all 5 treatments. However, there were also some IBOs that appeared in only one treatment, which we called unique IBOs (UIBOs). Hsy-Cme contained the largest number of UIBOs (521), followed by Hsy (418), Hsy-Csa (383), Pgr (261) and Hsy-Gsp (257). In addition, the compositions of IBOs in Hsy-Gsp, Hsy-Cme, and Hsy-Csa were quite dissimilar from that in Hsy. In conclusion, the composition of internal bacteria changed greatly during host alternation.

### Internal bacterial community diversity

**Figure 2:**
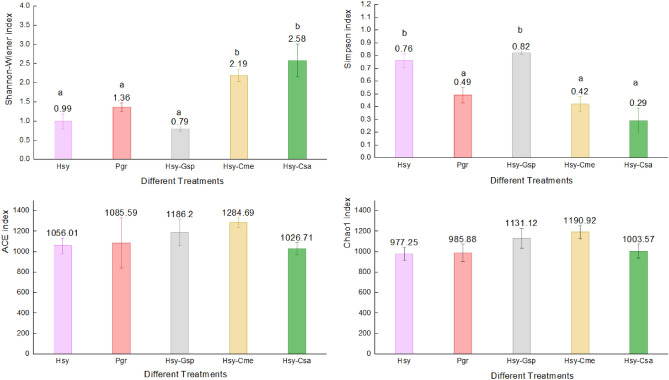
Bar chart of the Shannon–Wiener, Simpson, ACE and Chao1 indexes in Hsy, Pgr, Hsy-Gsp, Hsy-Cme and Hsy-Csa. The numbers above the bars represent the average of each index in different treatments. Different lowercase letters above the bars indicate significant differences in each index among treatments (P < 0.05).

The results of Shannon–Wiener, Simpson, ACE and Chao1 index analyses based on OTU classification are shown in Fig. 2. Shannon–Wiener and Simpson index analyses revealed that Hsy-Cme (Shannon–Wiener: 2.19 ± 0.16, Simpson: 0.42 ± 0.06) and Hsy-Csa (2.58 ± 0.42, 0.29 ± 0.10) had significantly higher diversity than Hsy (0.99 ± 0.19, 0.76 ± 0.05), Pgr (1.36 ± 0.12, 0.49 ± 0.06) and Hsy-Gsp (0.79 ± 0.05, 0.82 ± 0.01). These results indicated that when cotton aphids were transferred from hibiscus to different summer hosts, the diversity of their internal bacterial community (IBC) changed significantly. The number of OTUs increased significantly on cucumber and muskmelon, while that on cotton decreased slightly.

**Figure 3:**
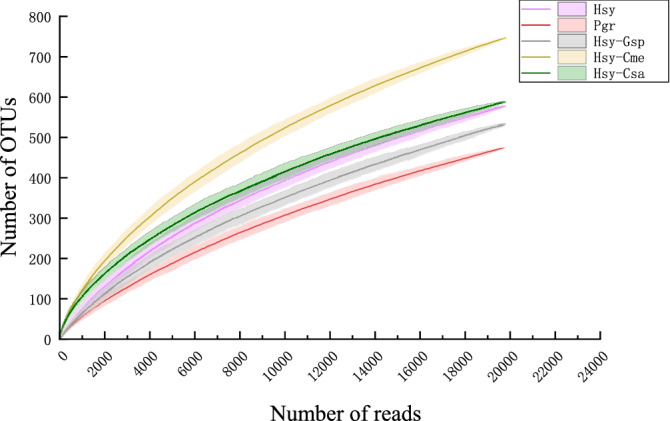
Rarefaction curves of cotton aphids on different host plants. Shaded areas represent 95% confidence intervals.

According to the ACE and Chao index results, the treatment with the highest richness index was Hsy-Cme (ACE: 1284.69 ± 46.34, Chao1: 1190.92 ± 63.37), followed by Hsy-Gsp (1186.2 ± 130.12, 1131.12 ± 96.91), Hsy (1056.01 ± 76.71, 977.25 ± 62.88) and Pgr (1085.59 ± 254.24, 985.88 ± 85.19). However, there were no significant differences among the treatments. These results indicated that the richness of the IBC in cotton aphids did not change much on different hosts but was relatively high on muskmelon. Rarefaction curves showed a trend similar to that of the species richness index, with the highest rarefied species richness in Hsy-Cme and the lowest in Pgr (Fig. 3).

**Figure 4:**
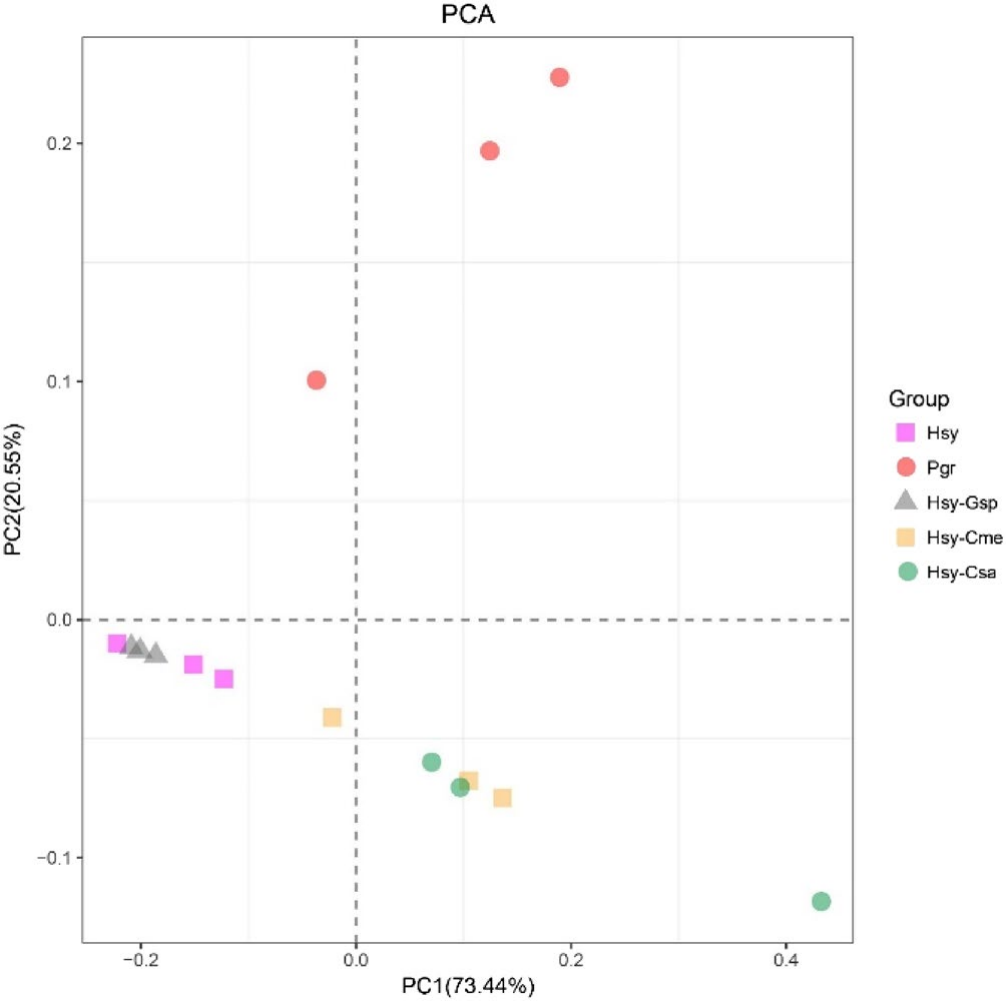
PCA of Hsy, Pgr, Hsy-Gsp, Hsy-Cme and Hsy-Csa. Each treatment is represented by 3 points corresponding to parallel experiments. Points with corresponding colors and shapes represent the same treatments.

The relationships among samples in terms of community composition were characterized with PCA (Fig. 4). The sum of the variation explained by the first two PCs, PC1 (73.44%) and PC2 (20.55%), reached 93.99%. This result indicated that there were two main factors influencing the change in the IBC during the host conversion process of cotton aphids. Additionally, the points of Hsy and Hsy-Gsp and the points of Hsy-Cme and Hsy-Csa were adjacent, and these two groups were mainly separated by the axis of PC1. In addition, the points of Pgr were far from those of the other groups on the PC2 axis.

### Relative abundance of IBCs among treatments

**Figure 5:**
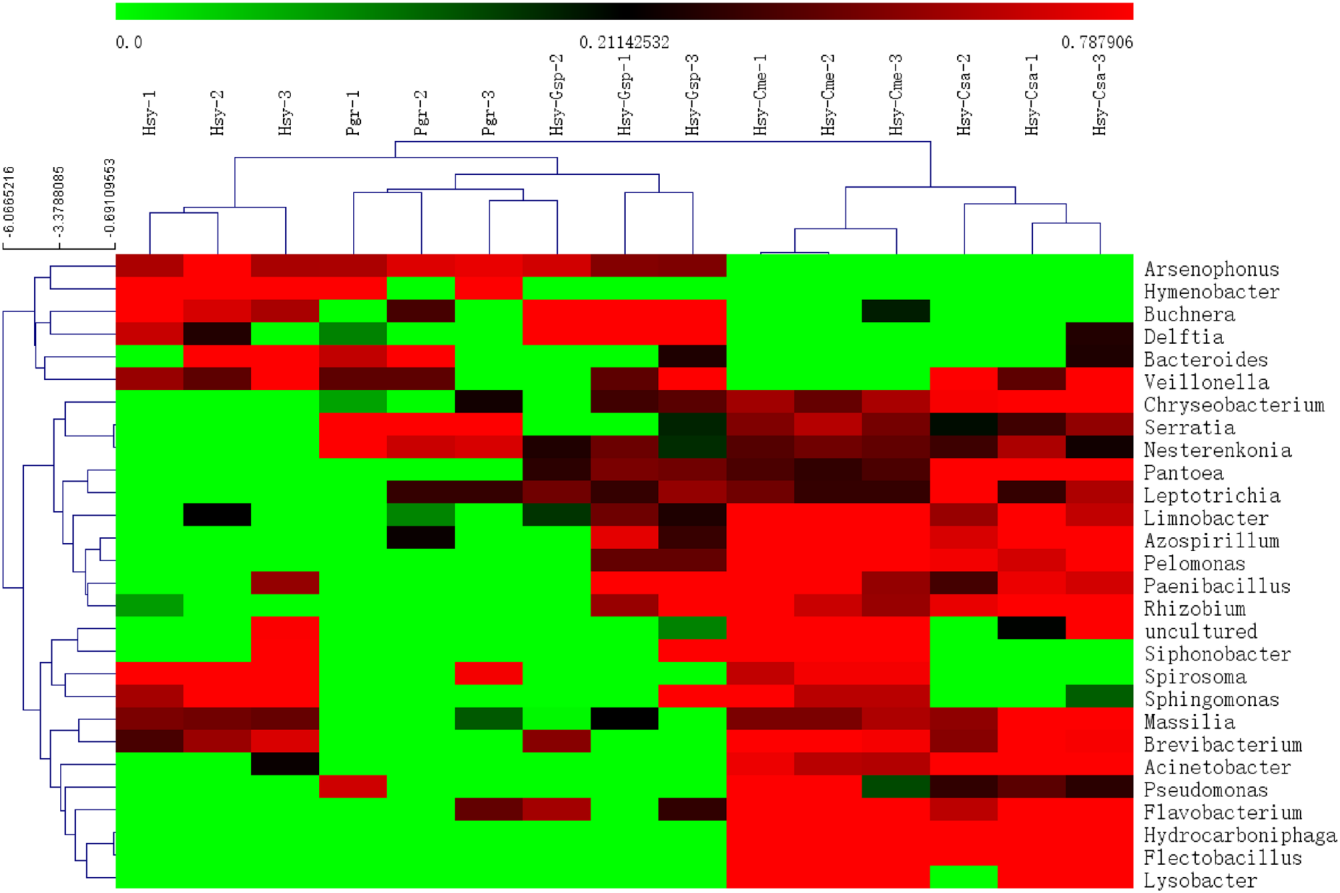
Heat map of the relative abundances (log_2_ transformed) of IBCs among Hsy, Pgr, Hsy-Gsp, Hsy-Cme and Hsy-Csa based on 16S rDNA gene Illumina sequencing. There were 3 parallel experiments in each treatment. The color gradient ranges from the minimum value in green to the maximum value in red. The expression values used were the Z value of normalized reads number. The clustering method of bacteria is HCL (Hierarchical Clustering). The sample tree was clustered with Euclidean Distance.

OTU data based on 16S rDNA were clustered at the phylum, class, order, family, genus and species levels, and the genus-level taxonomic results were used for follow-up analysis^24^. ANOVA was performed on the total abundance of the top 50 genera of IBCs in each treatment. The results revealed 28 genera in the IBCs with significant differences between two arbitrary treatments (P < 0.05). Genus- and sample-level clustering of those 28 differentially abundant genera in the IBCs are shown in the heat map (Fig. 5). The 3 replicates of each treatment were clustered into one branch, indicating the high reliability of our experimental data. The internal bacterial genera were grouped based on their relative abundances (log_2_ transformed) into 6 clusters. *Arsenophonus*, *Hymenobacter*, *Buchnera*, *Delftia*, *Bacteroides* and *Veillonella* clustered into one group and showed higher abundances in Hsy, Pgr and Hsy-Gsp than in Hsy-Cme and Hsy. *Chryseobacterium*, *Serratia* and *Nesterenkonia* formed another group, with higher abundances in Pgr, Hsy-Cme and Hsy-Csa than in the other treatments. *Pantoea*, *Leptotrichia*, *Limnobacter*, *Azospirillum*, *Pelomonas*, *Paenibacillus* and *Rhizobium* showed higher abundances in Hsy-Gsp, Hsy-Cme and Hsy-Csa than in the other treatments. The fourth group contained an uncultured bacterium, *Siphonobacter*, *Spirosoma* and *Sphingomonas*, whose abundances were higher in Hsy-Cme and Hsy than in the other treatments. *Massilia* and *Brevibacterium* formed an additional group. Their abundances were higher in Hsy, Hsy-Cme, and Hsy-Csa than in the other treatments. The last group, including *Acinetobacter*, *Pseudomonas*, *Flavobacterium*, *Hydrocarboniphaga*, *Flectobacillus* and *Lysobacter,* exhibited higher abundances in Hsy-Cme and Hsy-Csa than in the other treatments.

### Comparison of several common endosymbiotic bacteria in aphids among treatments

The obligate endosymbiont *Buchnera* and 3 facultative endosymbionts *Serratia*, *Arsenophonus* and *Wolbachia* were detected in our study. *Buchnera* and *Arsenophonus* appeared in all treatments, while *Wolbachia* and *Serratia* were found in only some of the treatments. *Wolbachia* was detected only in Hsy, whereas *Serratia* was detected in all treatments except Hsy (Table 1).

**Table 1:** Abundance of 4 common endosymbiotic bacteria (mean ± SE) in aphids among Hsy, Pgr, Hsy-Gsp, Hsy-Cme and Hsy-Csa.

	*Buchnera*	*Serratia*	*Arsenophonus*	*Wolbachia*
Hsy	17,719.67 ± 569.44bc	0.00 ± 0.00a	382.00 ± 115.02b	2.33 ± 1.45
Pgr	13,474.33 ± 1221.87ab	4684.00 ± 970.02b	394.33 ± 64.87b	0.00 ± 0.00
Hsy-Gsp	18,533.00 ± 131.11c	23.00 ± 11.00a	248.67 ± 68.25ab	0.00 ± 0.00
Hsy-Cme	13,013.67 ± 969.75ab	475.67 ± 145.88a	1.67 ± 0.33a	0.00 ± 0.00
Hsy-Csa	10,422.67 ± 2304.71a	214.33 ± 122.03a	2.00 ± 1.00a	0.00 ± 0.00
*P* Value	0.005	0.000	0.003	0.102

Different lowercase letters within a row represent significant differences among treatments (P < 0.05).

The abundance of the obligate endosymbiont *Buchnera* varied significantly among treatments, with Hsy-Gsp containing the highest abundance, followed by Hsy, Pgr, Hsy-Cme and Hsy-Csa. The proportion of *Buchnera* in all IBCs showed the same trend (Table 2). There were significant differences in the abundances and proportions of secondary endosymbionts in cotton aphids among treatments (Tables 1, 2). Pgr contained the highest abundance of *Serratia*, accounting for more than 20% of the endosymbionts. The proportions of *Serratia* in Hsy-Cme and Hsy-Csa both exceeded 1%. Only a small proportion of *Serratia* appeared in Hsy-Gsp (0.1%). *Arsenophonus* accounted for approximately 1% of the endosymbionts in Hsy, Pgr and Hsy-Gsp but only a small proportion (0.01%) in Hsy-Cme and Hsy-Csa. In addition, only a small proportion of *Wolbachia* (0.01%) was found in Hsy (Table 2).

**Table 2:** The proportions of 4 common and 3 probable symbiotic bacteria in aphids among Hsy, Pgr, Hsy-Gsp, Hsy-Cme and Hsy-Csa.

	Hsy(%)	Pgr(%)	Hsy-Gsp(%)	Hsy-Cme(%)	Hsy-Csa(%)
*Buchnera*	89.43	68.01	93.54	65.68	52.61
*Serratia*	0.00	23.64	0.12	2.40	1.08
*Arsenophonus*	1.93	1.99	1.26	0.01	0.01
*Wolbachia*	0.01	0.00	0.00	0.00	0.00
*Acinetobacter*	0.08	0.03	0.03	1.24	4.42
*Pantoea*	0.00	0.01	0.12	0.08	5.15
*Flavobacterium*	0.00	0.00	0.01	0.69	0.08
Others	8.54	6.33	4.92	29.89	36.65

*Acinetobacter*, *Pantoea* and *Flavobacterium* have been confirmed as symbiotic bacteria in other insects. The abundance of *Acinetobacter* was significantly higher in Hsy-Cme and Hsy-Csa than in the other treatments (*P* < 0.001). The abundance of *Pantoea* was significantly higher in Hsy-Csa than in the other treatments (*P* = 0.004). The abundance of *Flavobacterium* was significantly higher in Hsy-Cme than in the other treatments (*P* < 0.001) (Table 3).

**Table 3:** Abundance of 3 symbiotic bacteria in insects (mean ± SE) among Hsy, Pgr, Hsy-Gsp, Hsy-Cme and Hsy-Csa.

	*Acinetobacter*	*Pantoea*	*Flavobacterium*
Hsy	16.00 ± 13.01a	0.33 ± 0.33a	0.00 ± 0.00a
Pgr	5.33 ± 0.88a	1.00 ± 0.58a	0.67 ± 0.67a
Hsy-Gsp	6.00 ± 5.03a	24.67 ± 6.44a	2.00 ± 1.53a
Hsy-Cme	246.67 ± 44.81b	16.33 ± 1.67a	137.00 ± 31.63b
Hsy-Csa	876.67 ± 90.72c	1020.00 ± 363.96b	16.33 ± 4.98a
*P* Value	0.000	0.004	0.000

Different lowercase letters within a row represent significant differences among treatments (P < 0.05).

## Discussion

The cotton aphids in our study were collected from hibiscus and pomegranate in fields and cotton, muskmelon and cucumber in the laboratory. The insects sampled from the latter group of hosts were transferred from hibiscus and established a stable population with over 10 generations of cultivation. There were apparent differences in the OTU-based community composition of internal bacteria among the 5 treatments. The IBOs shared between pairs of treatments accounted for less than half of the total IBOs, indicating that the IBOs of aphids changed greatly among host plants. The Shannon–Wiener index and Simpson index showed that the community diversities were significantly higher in cotton aphids on muskmelon and cucumber than in those on the other plants (Fig. 2). In addition, the species richness on muskmelon and cucumber was higher than that on the other plants, based on the rarefaction curves. PCA revealed two PCs affecting the change in IBOs during host alternation (Fig. 4). We speculate that the two main reasons might be related to the differences in phloem sap components among plants in different families. During experiment section, cotton aphids on hibiscus and pomegranate were all transferred to cotton, muskmelon and cucumber respectively. However, only the aphids from hibiscus could survive on cotton, muskmelon and cucumber. This phenomenon was consistent to the PCA results and the rarefaction curves that the microbiome communities of the aphids on pomegranate were quite different from others.

One obligate endosymbiont (*Buchnera aphidicola*) and 9 facultative endosymbionts (*Serratia symbiotica*, *Wolbachia*, *Arsenophonus*, *Hamiltonella defensa*, *Regiella insecticola*, *Rickettsia*, *Rickettsiella*, Fukatsuia and *Spiroplasma*) have been described in aphids to date^25,26^. In our study, *Buchnera* and 3 facultative endosymbionts of aphids (*Serratia*, *Arsenophonus* and *Wolbachia*) were detected. In addition, we found 3 bacteria (*Acinetobacter*, *Pantoea* and *Flavobacterium*) that have been previously reported as symbionts in insects (Table 3).

The obligate endosymbiont (*Buchnera*) occupied the most important position. In addition, the proportion of this endosymbiont exceeded 52% in all treatments. The percentage of *Buchnera* in cotton aphids was as high as 93%. Zhao et al. found that the abundance of *Buchnera* in cotton aphids in China was between 72 and 95%, which was similar to our result^27^. Based on isotope-containing nutrient solution feeding, high-temperature stress treatment and antibiotic feeding, the main function of *Buchnera* was found to be the provision of a variety of essential amino acids to host aphids^28,29^. The genomic research indicated that *Buchnera* can provide the biosyntheses of amino acids essential for the hosts. And they lacks cell-denfence genes. *Buchnera* is completely symbiotic^30^.

The abundance of *Serratia* in cotton aphids fed pomegranate was significantly higher than that in the other four treatments. *Serratia* was not found in Hsy. However, after aphids were transferred to cotton, cucumber and muskmelon, *Serratia* was detected (Table 2). Currently, *Serratia* is known to be involved in the defense against various adverse conditions of its host aphids. For instance, this endosymbiont increases the resistance of aphids to parasitoid wasps^31^ and the ability of aphids to withstand high temperatures^32^. The abundance of *Serratia* increased as the aphids grew^33^ and was greater in summer than in collections made 2–4 months earlier^34^. In addition, there was some evidence that *Serratia* can supply nutrients. This process might be involved in the generation of tryptophan^35^. Studies of *Cinara cedri*^36^ and *Tuberolachnus salignus*^37^ revealed a convergent split of the tryptophan biosynthetic role between *Buchnera* and *Serratia*. Serratia is a co-obligate symbionts in these specific aphids. Serratia also have been shown to be involved in B vitamins bio synthesis in Lachninae^38,39^. In our study, the proportion of *Serratia* in Pgr reached 24%. The proportion was so high that facultative endosymbionts were rarely observed. Additionally, the abundance of *Serratia* on the 5 host plants was in contrast to the abundance of *Buchnera*. Wilkinson et al. found that *Serratia* was inversely related to the nitrogen content of food, in contrast to *Buchnera*^28^. Thus, *Serratia* and *Buchnera* may play compensatory roles. Cotton aphids on pomegranate may rely more on *Serratia* for its amino acid assimilation. Nevertheless, a further study needs to be conducted.

*Arsenophonus* widely infects many insects, including aphids, and other organisms^40,41^. In our study, *Arsenophonus* was detected in all samples (Table 3). It was significantly less abundant on the host plants of *Cucurbitaceae* (muskmelon and cucumber) than on the other host plants. Recent studies showed that coinfection by *Arsenophonus* and *Hamiltonella* enhances the fitness of cotton aphid^42^. *Arsenophonus* and *Wolbachia* may produce B vitamins^43^. In addition, *Arsenophonus* can mediate the host specialization of several herbivores. For instance, *Arsenophonus*-infected *Aphis craccivora* was found to specialize on locust tree, while uninfected clones appeared to specialize on alfalfa^44^. Therefore, in our treatments, *Arsenophonus* might have been associated with the specialization of cotton aphids on Hsy and Pgr by enhancing the fitness of cotton aphid in the wild or complementing the nutritional metabolism of the host aphids.

*Wolbachia* has been found in a variety of arthropods and is usually associated with their reproduction. This effect allows arthropods to reach high abundances in a short period of time^45^. However, *Wolbachia* is considered to have a low prevalence in aphids. Augustinos et al. reported that 37 of 425 aphid samples were infected by *Wolbachia*^46^. We detected only a small proportion of *Wolbachia* (0.01%) in Hsy. Similarly, Liu et al. and Zhao et al. found only a very small number of *Wolbachia* in cotton aphids throughout China^27,47^. In addition to the low abundance of *Wolbachia*, this phenomenon may be due to the difficulty in detecting *Wolbachia* in aphids. PCR detection of *Wolbachia* is limited^46^. In addition, *Wolbachia* genes can be transferred to the host genome, making *Wolbachia* detection even more difficult^48^.

*Acinetobacter*, *Pantoea* and *Flavobacterium* are three symbiotic bacteria that have been reported in insects, excluding aphids. *Acinetobacter* and *Pantoea* were found in both the Hessian fly (*Mayetiola destructor*) and samples of its host (wheat) that were infested and susceptible to the Hessian fly, suggesting that *Acinetobacter* and *Pantoea* play an essential role in Hessian fly-wheat interactions^49^. The gut bacteria of beetles*,* such as *Acinetobacter,* have been linked to the expansion of the diet breath of these beetles on exotic plants^50^. In our study, *Acinetobacter* and *Flavobacterium* were significantly more abundant in Hsy-Cme and Hsy-Csa than in the other treatments. And Acinetobacter and Pantoea were significantly more abundant in Hsy-Cme and Hsy-Csa than in the other treatments. Therefore, we suppose that the function of these bacteria may be associated with the expansion of cotton aphids’ host plants and these bacteria’ coinfection of aphids and host plants may make the host plants more susceptible. Therefore, in future research, we shall detect the existence and abundance of these bacteria on corresponding host plants. In addition, *Flavobacterium* was found to act as a male-killing bacterium in Japanese ladybirds, such as *Coccinula crotchi* and *Coccinula sinensis*. Environmental conditions may affect the distribution and spread of *Flavobacterium*^51^. This bacterium had a significantly higher abundance in the aphids on host plants of hibiscus, pomegranate and cotton than in those on other host plants. The abundance of *Flavobacterium* increased after aphids were transferred to muskmelon and cucumber as host plants. Therefore, the parthenogenetic cotton aphid male-killing effect on muskmelon and cucumber might be caused by *Flavobacterium* due to environmental changes. *Arsenophonus* has been confirmed to have the male-killing function^52^. However, this function of *Arsenophonus* and *Flavobacterium* has not been confirmed in aphids.

Symbiotic bacteria play an essential role in the specialization of aphids to their host plants. Some specific symbiotic bacteria can correspondingly enhance the fitness of aphids on their specific host plants; for example, the fitness of *Acyrthosiphon pisum* on specific plants was enhanced by *Regiella*^53,54^. In this study, *Serratia* was favored on pomegranate due to its amino acid assimilation or some other form of nutrient metabolism. The abundance of *Arsenophonus* was deduced after transferring aphids from hibiscus to cucumber and muskmelon, which may also be associated with the specialization of cotton aphids. In addition, *Buchnera*, *Acinetobacter*, *Pantoea*, *Flavobacterium* and other genera all varied greatly among the 5 treatments in this experiment. There may be some correspondence between host plants and specific composition of symbiotic bacteria, like the different abundance distribution of symbionts that showed in Fig. 5. In subsequent studies, more relevant plants will be included. Further research on the mechanism of host alternation will also be conducted.

## Limitation of the study

We just used universal primers to amplify the sequences of bacterial. Although the characteristics of bacterial community can be explained to some extent, the preference of amplification may lead to some deviation.

## Methods

### Plants and aphid

All host plants were conventional strains planted in China. The winter host plants included hibiscus (*Hibiscus syriacus*) and pomegranate (*Punica granatum*), which were planted on the campus of Minzu University of China in Haidian District, Beijing. The sampling location of cotton aphids from hibiscus and pomegranate are right on either side of a 5-m wide road. The summer host plants included cotton (*Gossypium* spp., Zhongmian 16), muskmelon (*Cucumis melo*, Japanese melon), and cucumber (*Cucumis sativus*, Jinqingxinyan 4) and were all cultured in cylindrical pots with a height of 9.5 cm and a diameter of 9.5 cm. The soil used for cultivation consisted of vermiculite and nutrient soil. Before planting, the soil was well mixed and sterilized at 180 °C for 2 h. The host plants were cultured in an incubator to a suitable stage for cotton aphid infection.

The cotton aphids on hibiscus and pomegranate were collected in spring, aphids from different host species is a sample. 20 adult aphids were transferred to a centrifuge tube containing absolute ethanol as a replicate. These tubes were marked as Hsy (cotton aphids on hibiscus) and Pgr (cotton aphids on pomegranate) and stored at − 20 °C. Each sample has 3 replicates from 3 hibiscuses and pomegranates. The distance between the trees was 5 m. At the same time, cotton aphids on hibiscus were transferred to three different summer host, each host transferred 50 and this process was repeated three times as replicates. After 24 h, those 50 cotton aphids were removed and only offsprings were remained on summer hosts. After more than 10 generations of parthenogenesis, the offsprings had established stable populations on cotton, muskmelon and cucumber. Then, 20 adult aphids were collected into tubes containing absolute ethanol as a replicate. These tubes were labeled as Hsy-Gsp (cotton aphids on cotton transferred from hibiscus), Hsy-Cme (cotton aphids on muskmelon transferred from hibiscus) and Hsy-Csa (cotton aphids on cucumber transferred from hibiscus) and stored at − 20 °C. All samples were independently transferred from hibiscus to cotton, muskmelon and cucumber. Cotton aphids on pomegranate were also transferred to cotton, muskmelon and cucumber. However, theses aphids transferred from pomegranate couldn’t establish stable populations.

The cultivation temperature was 27 °C during the day and 25 °C at night. The photoperiod was 16 L: 8 D. The light intensity was 3000 to 4000 lx. Each replicate was placed in a 40 cm × 40 cm gauze cage, separate from other replicates. A tray was placed at the bottom of the cage to maintain moisture and humidity.

### DNA extraction

The collected adult aphids were surface sterilized with absolute ethanol. Then, we used sterile water to rinse off the remaining ethanol. Finally, aphids were air dried on a clean bench. After air drying, 2 mg of adults (≤ 20 adult aphids) was taken from each replicate for DNA extraction. DNA extractions were performed using a QIAamp DNA Stool Mini Kit (50) (QIAGEN, Germany) according to the manufacturer’s instructions. The purity and concentration of DNA were confirmed using 1% agarose gel electrophoresis and an ultraviolet–visible spectrophotometer (NanoDrop 2000, Thermo Scientific).

### Generation and sequencing of 16S amplicons

The primers used for aphid internal bacterial 16S rDNA amplification were 16S-F 5′-TCGTCGGCAGCGTCAGATGTGTATAAGAGACAGCCTACGGGNGGCWGCAG-3′ and 16S-R 5′-GTCTCGTGGGCTCGGAGATGTGTATAAGAGACAGGACTACHVGGGTATCTAATCC-3′^55^. Amplicons were generated in 25 μL volumes using 12.5 μL of 2 × KAPA HiFi HotStart ReadyMix (KAPA Biosystems, part # KK2601) containing 1 μM forward and reverse primers and 12.5 ng of template DNA. The reaction conditions for 16S amplification were as follows: 95 °C for 3 min; 25 cycles of 95 °C for 30 s, 55 °C for 30 s, and 72 °C for 30 s; and a final extension of 72 °C for 5 min. Dual indexes and Illumina sequencing adapters were attached to 16S amplicons with 8 additional cycles of PCR using a Nextera XT Index Kit (Illumina, catalog # FC‐131‐1001). Then, the products were sequenced on an Illumina MiSeq system using 2 × 300 bp paired-end multiplex sequencing. The generation of amplicons and sequencing of products were completed by Shanghai Biotechnology Corporation. The raw reads have been deposited in the NCBI Sequence Read Archive (SRA) database under BioProject accession number PRJNA591256.

### Sequence processing

The pair-ended sequence data were merged by FLASH v1.2.11. If the average quality score of reads were lower than 20, they will be trimmed in a 50-bp sliding window by Trimmomatic v0.36. The reads which were shorter than 50 bp after quality control will be filtered. Paired reads were merged into a sequence with the minimum overlap Length (10 bp) according to the relationship between overlap and reads. The maximum mismatching ratio was 0.2, and non-conforming sequences were screened. Distinguish samples and adjust the direction of the sequence according to the barcode and primer on both ends of the sequence. The maximum mismatching ratio of barcode was 0. The maximum mismatching ratio of primer was 2. The processing result was shown in Table S1.

### Statistical analysis

The denoised 16S rDNA sequences were clustered into operational taxonomic units (OTUs) based on 97% similarity using RDP Classifier v2.11. The blast database selected was the SILVA database (Release123: http://www.arb-silva.de)^56^. A representative sequence from each OTU was selected for downstream analysis. Venn diagrams were generated to visualize OTUs. OTU data were used to measure the community composition of each sample at each taxonomic level (phylum, class, order, family, genus and species). 16S rDNA sequences were then clustered at the genus level via MeV v4.9.0^57^, and expression values were used to generate a heat map.

Diversity and richness indexes, including the Shannon–Wiener index, Simpson index, abundance-based coverage estimator (ACE) index and Chao1 index, were calculated with mothur v1.36.1. A species rarefaction curve was constructed by Estimate S v9.1.0^58^. Principal component analysis (PCA) was performed to analyze the similarity of bacterial community structures (R v3.2.5). All data analysis and visualization were performed by using SPSS 23.0 and Origin 2019 v9.60. One-way ANOVA was used to detect differences between groups. All results are presented as the mean ± standard error (SE). When the *P* value was less than 0.05, the difference was considered statistically significant.

## Supplementary Information

Supplementary Information.

## Data Availability

Further information and requests for resources and reagents should be directed to and will be fulfilled by the Lead Contact, Yanjie Ma (17301114@muc.edu.cn).

## Data and code availability

The raw 16S rDNA reads during this study are available at the NCBI Sequence Read Archive (SRA) database under BioProject accession number PRJNA591256 (https://www.ncbi.nlm.nih.gov/bioproject/PRJNA591256).
