# Supplementary material for: Microbiome diversity of cotton aphids (Aphis gossypii) is associated with host alternation
Source: Sci Rep. 2021 Mar 4;11:5260. doi: 10.1038/s41598-021-83675-2 (PMC7933357; doi:10.1038/s41598-021-83675-2)
Supplement: Supplementary file 1 — Supplementary Information. [file 41598_2021_83675_MOESM1_ESM.docx]

*Supplementary Material*

Microbiome diversity of cotton aphids (*Aphis gossypii*) is associated with host alternation

Yan-jie Ma ^1, a^, Hao-peng He^1,^ ^a^, Hai-meng Zhao ^1^, Yi-dan Xian ^1^, Hui Guo ^1^, Biao Liu ^2^, Kun Xue^1,2, *^

^1^ College of Life and Environmental Sciences, Minzu University of China，Beijing100081, China

^2^ Nanjing Institute of Environmental Sciences, MEP, Nanjing 210042, China

^a^ These authors share the first authorship

**Table of contents**

Clean reads statistics S1

Raw Sequences of 16S rDNA of Cotton Aphids S2

The raw reads have been deposited in the NCBI Sequence Read Archive (SRA) database under BioProject accession number PRJNA591256. The correspondence between treatments and filenames is shown in the Table S2. Raw data can be downloaded from <https://www.ncbi.nlm.nih.gov/bioproject/PRJNA591256>

Table S1 Clean reads statistics

| Sample Name | Raw reads | Valid reads | Valid ratio(%) |
| --- | --- | --- | --- |
| Hsy-1 | 32,494 | 32,308 | 99.43% |
| Hsy-2 | 33,640 | 33,397 | 99.28% |
| Hsy-3 | 38,400 | 38,162 | 99.38% |
| Pgr-1 | 22,087 | 21,938 | 99.33% |
| Pgr-2 | 25,705 | 25,487 | 99.15% |
| Pgr-3 | 40,263 | 39,965 | 99.26% |
| Hsy-Gsp-1 | 29,884 | 29,653 | 99.23% |
| Hsy-Gsp-2 | 30,798 | 30,635 | 99.47% |
| Hsy-Gsp-3 | 29,638 | 29,423 | 99.27% |
| Hsy-Cme-1 | 39,978 | 39,690 | 99.28% |
| Hsy-Cme-2 | 36,876 | 36,587 | 99.22% |
| Hsy-Cme-3 | 26,475 | 26,261 | 99.19% |
| Hsy-Csa-1 | 39,481 | 39,227 | 99.36% |
| Hsy-Csa-2 | 46,245 | 45,899 | 99.25% |
| Hsy-Csa-3 | 40,764 | 40,495 | 99.34% |

| Table S2 Raw Sequences of 16S rDNA of Cotton Aphids | |
| --- | --- |
| Treatment | Filename |
| Collected from hibicus | 16S rDNA sequence of Aphis Gossypii: adult aphids on Hibiscus syriacus1 |
|  | 16S rDNA sequence of Aphis Gossypii: adult aphids on Hibiscus syriacus3 |
|  | 16S rDNA sequence of Aphis Gossypii: adult aphids on Hibiscus syriacus3 |
| Collected from pomegranate | 16S rDNA sequence of Aphis Gossypii: adult aphids on Punica granatum1 |
|  | 16S rDNA sequence of Aphis Gossypii: adult aphids on Punica granatum2 |
|  | 16S rDNA sequence of Aphis Gossypii: adult aphids on Punica granatum3 |
| Transffered from hibicus to cotton | 16S rDNA sequence of Aphis Gossypii: adult aphids on Gossypium spp.1 |
|  | 16S rDNA sequence of Aphis Gossypii: adult aphids on Gossypium spp.2 |
|  | 16S rDNA sequence of Aphis Gossypii: adult aphids on Gossypium spp.3 |
| Transffered from hibicus to muskmelon | 16S rDNA sequence of Aphis Gossypii: adult aphids on Cucumis melo1 |
|  | 16S rDNA sequence of Aphis Gossypii: adult aphids on Cucumis melo2 |
|  | 16S rDNA sequence of Aphis Gossypii: adult aphids on Cucumis melo3 |
| Transffered from hibicus to cucumber | 16S rDNA sequence of Aphis Gossypii: adult aphids on Cucumis sativus1 |
|  | 16S rDNA sequence of Aphis Gossypii: adult aphids on Cucumis sativus2 |
|  | 16S rDNA sequence of Aphis Gossypii: adult aphids on Cucumis sativus3 |
